# 2025 Conference on Bacteriophages: Biology, Dynamics, and Therapeutics

**DOI:** 10.20411/pai.v11i1.942

**Published:** 2026-02-09

**Authors:** Ahmed Elshazly, Daniel A. Russell, Graham F. Hatfull, Robert T. Schooley, Paul L. Bollyky, Julie D. Pourtois, Krista G. Freeman

**Affiliations:** 1 Division of Infectious Diseases and Global Public Health, Department of Medicine, University of California San Diego, San Diego, California; 2 Department of Biological Sciences, University of Pittsburgh, Pittsburgh, Pennsylvania; 3 Division of Infectious Diseases and Geographic Medicine, Department of Medicine, Stanford University School of Medicine, Stanford, California; 4 Biology Department, University of Maryland, College Park, College Park, Maryland and University of Maryland Institute for Health Computing, North Bethesda, Maryland; 5 Department of Physics, Case Western Reserve University, Cleveland, Ohio

**Keywords:** Bacteriophage, Phage, Antimicrobial Resistant Infections (AMR), Antibiotic Resistance, Phage Engineering, Synthetic Biology

## Abstract

The 2025 Conference on Bacteriophages: Biology, Dynamics, and Therapeutics, supported by the International Antiviral Society–USA, brought over 250 researchers, clinicians, and industry innovators from 17 countries to Washington, DC, from October 12 – 14, 2025. The meeting emphasized collaboration across the full spectrum of phage science—from molecular biology to clinical translation—reflecting a field rapidly translating novel biological insights from the laboratory into clinical applications. This summary provides highlights of the 43 oral and 97 poster presentations made during this 2.5-day conference.

## INTRODUCTION

Bacteriophages are hot! Hundreds of people with multidrug-resistant bacterial infections and infections of implanted devices are now receiving bacteriophage therapy yearly in the United States (US) and Europe on an expanded access basis under the supervision of the US Food and Drug Administration (FDA) and European regulatory agencies. Interest in phages has grown greatly over the past few years, fueled by 2 major developments. First, there is the recognition of the richness of bacterial systems that provide defense from phage attack, with hundreds if not thousands of newly identified systems that take us far past the better-known restriction-modification and CRISPR-Cas defenses. Together with phage-encoded anti-defense systems, these are dominant determinants in microbial evolution and profoundly shape microbial populations.

Second, there is renewed interest in the therapeutic potential of phages, especially in light of concerns about the growth in antibiotic resistance of bacterial pathogens and a very thin pipeline of new antibiotics and alternative approaches. Advances in both these areas require substantial advances in our understanding of phage biology and clinical studies to evaluate the therapeutic options.

The communities of scientists and clinicians sometimes, but not always, work closely to synergize their efforts. However, there is a clear need to more fully integrate these activities, with clinicians appreciating how studies in basic biology can advance translational studies and those working on phage fundamentals appreciating how their insights can advance clinical usage. These needs mirror prior experiences in our research fields, most notably in the early days of AIDS research when there was a need to understand HIV biology to develop diagnostic, preventative, and therapeutic solutions.

Further success in the development of phage therapeutics will require an integration of the rapidly growing appreciation of the complexity and elegance of phage biology with the principles of antimicrobial development that have been iteratively developed over the past 60 years. Critical gaps exist, in particular, in our understanding of phage pharmacokinetics and pharmacodynamics and in the rigor of *in vitro* techniques required to accurately predict phage efficacy in patients. Additional knowledge about the frequency, dynamics, implications, and mitigation of the development of phage resistance during therapy is also critical. The basic principles of phage therapeutics remain to be fully elucidated, but there are increasing expectations that clinical trials executed using microbiological and clinical endpoints that mirror those that have been validated in the development of small molecules will pave the way for widespread clinical use over the coming years.

The conference on *Bacteriophages: Biology, Dynamics, and Therapeutics* was designed to help integrate efforts in understanding phage biology with the challenges in clinical solutions. By bringing state-of-the-art microbiology together with leaders in clinical applications, we hope that new appreciations and new collaborations will stimulate the field of microbiology and address the public health challenges. Here, we provide a summary of the main activities in the conference including pre-meeting workshops, keynote and other oral presentations, and rich poster sessions.

## SUNDAY, OCTOBER 12, 2025

### Opening Remarks from the Chairs: Robert Schooley, MD, UC San Diego, and Graham Hatfull, PhD, University of Pittsburgh

Drs. Schooley and Hatfull opened the conference by highlighting the importance of integrating fundamental and translational research with clinical applications. They stressed that advancing phage therapy requires coordinated efforts across biology, manufacturing technology, clinical investigation, and clinical medicine. The chairs expressed enthusiasm for the strong presence of early-career investigators, whom they described as the driving force for the next generation of phage innovation. With 43 oral and 97 poster abstracts and tightly timed sessions, they encouraged attendees to engage actively, network widely, and use this conference as an opportunity to actively plan a research agenda that will provide novel insights into phage biology and accelerate the movement of these insights from the laboratory to the clinic.

### Opening Session: Evolution of Antiviral Immunity: From Phage Defense to Human Innate Response

Philip Kranzusch, PhD, Harvard University, opened the keynote session with a presentation entitled “Evolution of Antiviral Immunity.” Building on the insight that components of animal innate immunity originated billions of years ago in bacteria as phage defense systems, he hypothesized that the effect of eukaryotic immune proteins in bacterial cells could shed light on the mechanisms behind their antiviral function. In his keynote address, he presented unpublished results on the impact of eukaryotic immune defence molecules on phage predation in the bacterium *Escherichia coli*, focusing on the human protein SPRY domain-containing SOCS box protein 1 (SPSB1) and its effect on phage T7. He showed that SBSP1 inhibits the T7 replisome by binding to the gp4 primase domain of phage T7, recognizing a DxNxN loop motif that is also present in animal viral targets. This research highlights how the evolutionary connection between eukaryotic innate immunity and phage defenses can help explain mechanisms of immune signaling from bacteria to humans.

### Bench to Bedside: Phage Therapy in the United States

Daria Van Tyne, PhD, University of Pittsburgh, summarized the University’s extensive experience with Single Patient Investigational New Drugs (IND). Of nearly 900 requests, active phages were found for 323 cases; 106 received FDA approval, and 102 patients were treated—mostly intravenously—with excellent safety outcomes. Over half improved clinically, including several complete recoveries. Integrating pharmacokinetic and microbiome data, the team observed dynamic phage–bacteria interactions and occasional serum neutralization. Insights from these cases are now shaping ongoing clinical trials, including one for *Achromobacter* (opening 2026) and another for *Pseudomonas*. Their approach exemplifies real-time translation of discovery science into clinical care.

### Workshop 1: Clinical Trial Design & Regulatory Approaches

In addition to the 2 Opening Session Plenaries, 2 interactive workshops took place on the first day of the Conference. This first workshop provided insights into novel clinical trial designs and highlighted some of the practical considerations that complicate execution of clinical trials of phage therapeutics. The session closed with a presentation on key regulatory considerations by 2 key members of the FDA’s regulatory team.

### Considerations for Clinical Trials of Bacteriophages

Vance Fowler, MD, Duke University, discussed the critical need for novel therapeutic approaches in the treatment of antibiotic-resistant bacterial pathogens and laid out the rationale for the further investigation of phage therapy as a tool to this end. He pointed to similarities and differences in the antimicrobial, biological, and pharmacokinetic aspects of small-molecule antibiotics and bacteriophages and the implications of these features for clinical development. The challenges and opportunities related to the clinical development strategies focused on fixed vs personalized phage cocktails were outlined. The limitations of clinical trial designs in which phages are substituted for individual elements of an antimicrobial regimen and studied for non-inferiority were outlined and contrasted with “add-on” designs in which standard-of-care plus phage is compared to standard-of-care regimens in superiority endpoint trials.

### Practical Considerations for Clinical Trial Design and Analysis of Mycobacteriophage Treatment of NTM: The Design of the POSTSTAMP Trial

Jerry Nick, MD, National Jewish Health, outlined the challenges he faced in designing a 10-patient trial of bacteriophages for the treatment of non-tuberculous mycobacterial infections in people with cystic fibrosis. Challenges included the clinical heterogeneity of clinical trial participants and the comparability of control groups that will be composed of similar patients for whom lytic phages could not be found. Such challenges are not uncommon in cystic fibrosis trials.

### Regulatory Considerations and Data Update for Development and Clinical Use for Phage-Based Antimicrobials

Cara Fiore, PhD, and Timothy Brennan, MD, both of the Office of Vaccines Research and Review (OVRR), Center for Biologics Evaluation and Research (CBER), and FDA, outlined how phage therapy is regulated as a biologic under the FDA’s OVRR within the CBER. In the US, even single-patient compassionate uses require Single Patient IND submissions. While expanded-access INDs offer patient pathways, they cannot replace formal drug development requirements. Dr. Fiore described the FDA’s internal AI-based review tool, ELSA, which identifies common issues in Chemistry, Manufacturing, and Controls and trial design, such as incomplete genomic screening and inconsistent stability data. Dr. Brennan discussed trial design strategies, emphasizing staged safety evaluation, rigorous monitoring, and adaptive approaches for rare infections. Both presenters reinforced that phage therapy remains investigational, with the FDA committed to guiding safe, evidence-based advancement.

### Workshop 2: Preclinical Models of Phage Therapeutics

This workshop combined presentations on best practices, novel findings, and challenges associated with the use of preclinical *in vivo* models. Presentations highlighted how hypothesis-driven pre-clinical *in vitro* and animal model studies enhance our understanding of phage biology in eukaryotic hosts and can accelerate the clinical development of phage therapeutics.

### Mycobacteriophage in Animal Models

Sasha Larsen Akins, PhD, Seattle Children’s Research Institute, described her team’s use of murine pneumonia models to develop phage therapy cocktails to combat drug-resistant tuberculosis (TB). They found that aerosol phage therapy showed better lung coverage and persistence compared to intravenous (IV) administration. Phages were found to reduce TB burden in the lung and spleen, with aerosol phage therapy showing significant reductions in pathology. Their study highlighted the need for further research on phage efficacy, route of administration, and host immune responses and suggests that murine models can be an important component of elucidating those factors.

### Pulmonary Phage Therapy: Lessons From the Murine Model

Laurent Debarbieux, PhD, Institut Pasteur, shared his work on phage therapy in murine pneumonia models. His team has found that intratracheal phage dosing maintains high lung levels, whereas IV dosing results in transient levels. They identified a critical role for airway macrophages in phage clearance and antagonism of phage therapy, whereas neutrophils are essential to phage effectiveness. Their findings argue for pathogen- and host-context–specific strategies, potential phage engineering/formulation to evade clearance, and future personalized phage therapy guided by immunoprofiling.

### *Caenorhabditis elegans* as a Model System for Phage Biology

Pat Secor, PhD, University of Montana, presented the use of *C. elegans* to study host–microbe interactions and phage biology. The worm model has played key roles in elucidating the contributions made by Pf, a temperate phage that parasitizes *Pseudomonas aeruginosa*, to bacterial colonization and metabolism. The model is a powerful tool for pathogenesis and phage–host studies, though lytic phage therapy testing is confounded by loss of the worms’ food source. Dr. Secor also emphasized the importance of using multiple, orthogonal models in different species to inform our understanding of phage-host interactions.

## MONDAY, OCTOBER 13, 2025

### Symposium: The Bacteriophage Landscape: Diversity and Evolution *In Vitro* and *In Vivo*

The second day of the meeting included a morning symposium on phage diversity and evolution, as well as 2 parallel oral abstract sessions. Each abstract session was introduced by an invited plenary presentation. Afternoon programming included a multidisciplinary symposium that aimed to explore how insights about phage biology could be exploited to advance the clinical utility of phage. In addition, there were 8 poster-driven abstract sessions at which 52 posters were presented.

### Diversity of Microviruses: What We Know and Don’t Know

Paul Kirchberger, PhD, Oklahoma State University, explored the overlooked world of microviruses—tiny single-stranded DNA phages abundant in the human gut. His lab uses metagenomics analyses to discover new microviruses and synthetic assembly to understand the functional roles of different regions of the phage genome. For example, he reported on a collaboration with structural biologists that connected the hypervariable region of microvirus genomes, which encodes for protrusions in the capsid protein, to structural diversity that determines host-range, finding that single amino acid changes can dramatically alter infection profiles. Kirchberger emphasized that microviruses’ small, plasmid-like genomes make them ideal for synthetic and therapeutic applications, though they remain underrepresented in research collections.

### Phage Evolution *In Vitro* and *In Vivo*

Paul Turner, PhD, Yale University, discussed evolutionary tradeoffs in phage–bacteria interactions and how these dynamics can be leveraged in therapy. He showed that resistance often carries fitness costs—such as loss of virulence or increased antibiotic susceptibility. His group’s work in cystic fibrosis patients infected with *P. aeruginosa* demonstrated that while phage resistance emerged, bacterial virulence decreased, leading to clinical improvement. Turner advocated for using evolution predictably—“phage steering”—to guide bacteria toward less harmful phenotypes.

### Prophages: The Hidden Defense Reservoir

Karen Maxwell, PhD, University of Toronto, highlighted how integrated prophages protect bacteria against secondary phage infection and enhance fitness. Her lab identified RIP1, a prophage-encoded protein that blocks incoming phage replication. Cryo-EM studies revealed an 11-subunit terminase complex capped by 12 Receptor-interacting protein 1 (RIP1) monomers, suggesting a pore-based defense mechanism. She concluded that prophages represent vast, underexplored reservoirs of bacterial immunity that shape microbial evolution and influence therapeutic outcomes.

### Oral Abstract Session: Bacteriophage Biology

### Invited Plenary: New Views of Phage Tail Assembly and Disassembly

The session was opened by a structure-driven overview presentation from Alan Davidson, PhD, University of Toronto, on siphophage tail tip complexes and related proteins. The phage lambda genome encodes gpK, which has a deubiquitinating domain, and, just downstream, gpI, a conserved structural protein with a ubiquitin-like domain on its N-terminus. Through a series of protein structure predictions, biochemistry experiments, and phage mutational analyses, his group showed that gpK cleaves the ubiquitin-like domain of gpI from its structural domain and that this interaction ensures the correct stepwise assembly by occurring only after gpI interacts with other proteins in the lambda tail tip complex. The second half of the talk focused on tailocins, phage tail-like proteins that kill bacteria by puncturing the inner membrane. With high resolution structural studies, he showed that tailocin binding to and penetration of the bacterial host is accompanied by a massive conformational change of the central tail fiber protein, from a high-energy coiled coil state to a low-energy beta prism. This fold switching phenomenon results in ejection of the tape measure protein, which punctures the cell. Such fold switching may be a conserved structural feature among about one-third of tailed bacteriophages.

### Oral Abstracts

Lucas Moriniere, PhD, University of California Berkeley, presented a high-throughput effort to determine, both experimentally and via *in silico* predictions, the interactions between host cell receptors and receptor-binding proteins of *E. coli* phages. Experiments show that many phages use a 2-step binding process that may involve outer membrane proteins like porins, the core sugars of lipopolysaccharide, surface polysaccharides, and more. These data helped build predictive models for each host receptor protein. Some have been experimentally validated.

Avery Noonan, PhD, Lawrence Berkeley National Laboratory, discussed a strategy to predict phage-host interactions in a phylogeny-independent manner using a machine-learning tool trained on 8 large datasets of phage-host interactions. For one phage-host pair, the tool identified 35 of the 51 gene regions experimentally shown to be important for phage fitness. With further development, the pipeline could be applied to phage cocktail design.

Jyot Antani, PhD, Yale University, highlighted a mixed experimental/computational approach to predicting interactions between phages and hosts. The experimental tools include efficiency of plating determination and microscopic measurement of phage-host binding strength, as well as swim and twitch diameters of phage-infected cells. Plotting these data against each other may reveal correlations that can be exploited to predict phage susceptibility of patient isolates.

Andres Valdez, PhD, Pennsylvania State University, shared insights from computational simulations of bacterial cell reordering in response to phage infection. By treating uninfected bacterial cells, phage-infected bacterial cells, and phages as distinct particles in large-scale simulations, plaque formation could be replicated.

Benjamin Adler, PhD, University of California Berkeley, discussed the development of CRISPRi-ART for high throughput evaluation of the role of phage proteins on fitness. CRISPRi-ART utilizes dCas13d to target RNA transcripts and blocks translation of specific phage proteins. This tool captures known fitness-associated phage proteins identified with traditional techniques and has the capacity to identify, at scale, essential genes and regions.

Narbada Upreti, PhD, Catholic University of America, used cryo-EM to structurally define the attachment of the bacteriophage T4 packaging motor protein to its portal. Each subunit of the motor protein consists of an N-terminal ATPase domain, C-terminal nuclease domain, and C-terminal hairpin. The hairpin motifs of the pentameric motor assembly bind to the clip domains of the dodecameric portal; mutations in this region disrupt DNA packaging.

Anat Herskovits, PhD, Tel Aviv University, described a new *Listeria monocytogenes* prophage-encoded defense system that blocks virion production. Alphafold predictions suggest the defense protein, terI, binds to the small terminase protein, disabling it and preventing DNA translocation through the terminase complex. The prophage genome also encodes 2 proteins that neutralize terI, providing self-immunity so normal virion assembly can occur during the lytic cycle.

### Oral Abstract Session: Clinical Applications of Bacteriophages

### Invited Plenary: Assessment of Phage Susceptibility Testing Reproducibility and Agreement Between Methods

Robin Patel, MD, Mayo Clinic, opened the session with an insightful miniplenary that detailed critical efforts to standardize phage susceptibility testing, a key step for clinical implementation. The European Committee for Antibiotic Susceptibility Testing (EUCAST) is supporting an effort to define reference methods, interpretive criteria, and multicenter reproducibility standards. Early results from inter-laboratory studies show strong agreement in liquid-based assays for *E. coli* and *Staphylococcus aureus*. Dr. Patel stressed that reproducibility must precede interpretive guidelines, much like early antibiotic testing, and that harmonized methods are essential for future clinical trials. As in the case of antibiotic susceptibility testing, the ultimate goal of this process is to develop laboratory methodologies that will predict microbiological activity in the clinic. This will require iterative efforts, a clinical correlation, and assay optimization. The success of this effort will be critical to the successful application of phage therapeutics.

### Oral Abstracts

### BX211 Phage Therapy Effective in Phase II Trial for *S. aureus* Diabetic Foot Osteomyelitis

Merav Bassan, PhD, BiomX, presented the results of a prospectively randomized and blinded Phase II study of 41 patients with diabetes mellitus and foot ulcers due to *S. aureus*. The trial randomized study participants in a 2:1 ratio comparing best available antibiotic plus phage therapy to best available antibiotics alone. The therapeutic intervention phage cocktail (BX211) was safe and well-tolerated. Entry criteria required that *S. aureus* be isolated from bone biopsies taken prior to trial entry. Phage recipients exhibited more rapid and sustained reduction in ulcer size. There was no difference in the degree of response between phage recipients harboring methicillin-susceptible or methicillin-resistant *S. aureus*.

### CRISPR-Cas3-Engineered Bacteriophage Cocktail Developed to Broadly Target *P. aeruginosa* Infections

Ashley Trama, PhD, LOCUS Biosciences, presented a pipeline that integrates CRISPR–Cas3 systems into engineered phages, guided by AI-based cocktail optimization. Her team’s 6-phage cocktail targets respiratory pathogens, with engineered phages achieving broader coverage and faster bacterial clearance than wild-type strains. In murine infection models, engineered constructs outperformed natural counterparts, showing enhanced lytic activity and safety. These innovations form a foundation for next-generation phage therapeutics advancing toward clinical trials.

### Phage Therapy of Patients With Cystic Fibrosis With *Achromobacter xylosoxidans:* 3 Case Studies

Anca Segall, PhD, San Diego State University, shared results from compassionate-use phage therapy in children with cystic fibrosis who were chronically infected with *Achromobacter xylosoxidans*. Across several cases, intravenous phage therapy was safe, reduced bacterial load in sputum, improved lung function, and restored partial antibiotic susceptibility. Metagenomic data showed a shift toward “healthier” airway microbiomes. She concluded that phage therapy is a feasible adjunct for pediatric cystic fibrosis, potentially delaying the need for lung transplantation.

### Phage Cocktails vs *Pseudomonas aeruginosa* Pulmonary Infection: Delivery Routes and Pharmacokinetics

Nino Mzhavia, PhD, Walter Reed Army Institute of Research, reported on their evaluation of a rationally designed phage cocktail targeting multidrug- and extensively drug-resistant *P. aeruginosa* in a mouse lung infection model. The cocktail, composed of phages from 3 genera covering diverse bacterial receptors, was tested via intranasal and intravenous administration. Intranasal delivery achieved far higher lung concentrations and faster bacterial clearance, with phage amplification and normalization of inflammatory cytokines by 14 hours. Intravenous delivery showed delayed but eventual phage amplification and bacterial reduction. Individual phages displayed distinct pharmacokinetics, with faster-lysing phages yielding better outcomes. Pretreatment also improved survival. Overall, they found that intranasal phage therapy demonstrated strong efficacy and represents a promising strategy for multidrug-resistant and extensively drug-resistant *Pseudomonas* lung infections.

### Phages Act as Immune Triggers in the Inflamed Gut but Can Be Rewired

Dwayne Roach, PhD, San Diego State University, showed evidence of a bacterial bloom following phage treatment of gut infections in mice. Disease exacerbation occurred in both immunocompetent and lymphoid-deficient mice but differed by route of exposure. Rectal delivery resulted in earlier phage detection in the distal gut than oral delivery and was associated with worse disease severity. These effects were hypothesized to result from lysis-derived bacterial debris and immunostimulatory phage components. Lytic phages that were engineered to evade innate immune recognition were found to retain bactericidal activity and exhibit reduced immunostimulatory potential. He proposed that phage DNA activates Toll-like receptor 9 (TLR9), driving inflammation. His lab is now developing “immune-silent” phages to minimize these responses. These findings point to potentially intriguing and poorly understood aspects of phage behavior in the gut.

### Bacterial Receptors but Not Antiphage Defense Mechanisms are Major Determinants of Phage Host Range

Julie Pourtois, PhD, Stanford University and the University of Maryland, presented work on identifying the genetic determinants of *P. aeruginosa* susceptibility to phage OMKO1. Using a transposon mutagenesis screen and a GWAS approach incorporating lytic plaque assays and 80 clinical isolates of *P. aeruginosa* from patients with cystic fibrosis, her team found that receptor structures were implicated in phage susceptibility. In particular, phage OMKO1 was found to use flagella to infect *P. aeruginosa*. Conversely, phage defense systems, as identified by Padlock, were not predictive of OMKO1 susceptibility. These data suggest that receptors and not defense elements predict host range for this phage.

### Optimization of Phage Cocktail Design and Administration by a Genetic Algorithm

Nicholas Smith, SUNY Buffalo, reported on pharmacodynamics-driven optimization strategies for a 3-phage cocktail design using a machine learning genetic algorithm. Bacterial killing was first simulated for 100 random phage cocktail regimens, and top regimens were then tested using a Hollow Fiber Infection Model, which can mimic *in vivo* concentrations and elimination dynamics. The final cocktail regimen optimized by the algorithm contained LUZ19, E215, and PYO2, with LUZ19 administered first, followed by E215 and PYO2, and was expected to lead to cyclic phases of growth and killing. Dynamics observed in HFIM experiments agreed with model predictions, highlighting the potential of pharmacokinetic/pharmacodynamic models to design more complex phage cocktails.

### Afternoon Symposium: Lessons from the Clinic: Exploiting Bacteriophage Biology to Enhance Success

### Phage-Induced Immunity

Austen Terwilliger, Tailor Labs at Baylor University, shared recent work on the role of phage-induced immunity in the efficacy of phage therapy. In animal models, phage treatment not only eliminated infection but also provided strong, long-term protection against reinfection—reducing bacterial burden by up to 1 billion-fold compared to controls. This protection depended on phage-mediated bacterial lysis but did not require continued phage presence or the presence of residual resistant bacteria. *In vitro* experiments showed that lysed bacteria alone offered partial protection, suggesting that immune stimulation triggered by bacterial lysis contributes to the effect. Their findings indicate that some phages may evoke adaptive host immunity, acting both as antimicrobials and as immunizing agents to prevent recurrent infections, effectively combining therapeutic- and vaccine-like mechanisms.

### Phages vs Biofilms: Advancing Phage Therapy for Complex Prosthetic Joint Infections due to Staphylococci

Tristan Ferry, MD, PhD, Université de Lyon, shared the PHAGEinLYON Clinic team’s recent experiences with compassionate phage therapy in the management of prosthetic joint infections, exploring its underlying mechanisms, clinical outcomes, and practical considerations. Dr. Ferry presented data from over 100 patients on the efficacy of phage therapy against biofilms and also discussed other technical advances in phage therapy technology, including combination therapies with antibiotics. He highlighted the need for phages to treat biofilms because few antibiotics are active against biofilms, and they are highly toxic. Furthermore, he addressed the future potential of phage therapy to be integrated into standard treatment protocols for management of prosthetic joint infections, focusing on ongoing innovations and research areas. Building on this work, they launched Europe’s first randomized clinical trial testing intraoperative phage therapy for prosthetic joint infections.

### Innovating Phage Therapy for Respiratory Infections: Delivery Methods and Sequential Phage Applications for *P. aeruginosa*

Jonathan Koff, MD, Yale University, discussed personalized nebulized phage therapy for chronic *Pseudomonas* infections in cystic fibrosis. Among 9 patients, short phage courses improved lung function and reduced bacterial titers in sputum. Resistance-associated tradeoffs included reduced bacterial virulence and increased antibiotic susceptibility, underscoring phage therapy’s evolutionary potential.

## TUESDAY, OCTOBER 14, 2025

### Morning Symposium: Bacteriophage Resistance, Synergy, Tradeoffs: Mechanisms and Consequences

### Exploiting the Features of Mobile Genetic Elements to Identify Antiphage Defenses

Breck Duerkop, PhD, University of Colorado, examined how mobile genetic elements, particularly plasmids in *Enterococcus faecalis*, encode defense genes that block phage infection. His group identified two genes on plasmid pTEF2 that provide antiphage protection, emphasizing the evolutionary interplay between mobile elements and phage resistance.

### Fellowship of the Phage: The Interplay of Microbial Communities and Bacteriophages

Megan Castledine, PhD, University of Exeter, discussed how ecological context influences phage resistance. In complex microbial communities, resistance evolves more slowly and often carries fitness costs. She noted that clinical settings mirror this complexity, making it crucial to interpret laboratory findings within realistic ecological frameworks.

### Mapping the Landscape of Antiphage Defense Systems

Michael Laub, PhD, Massachusetts Institute of Technology, described the use of large-scale genetic screens and a machine-learning tool, Defense Predictor, to uncover thousands of new bacterial defense systems. Many were found in prophages, suggesting that bacterial genomes devote extensive resources to antiviral defense. His talk highlighted the growing role of AI in mapping microbial immunity.

### Oral Abstract Session: Bacteriophage Structure, Synthesis, and Engineering

### Miniplenary: Integrating Phage Biology and Therapeutics

Martha Clokie, PhD, University of Leicester, discussed the importance of considering a phage in its environmental context when designing a therapeutic regimen. She emphasized bridging phage biology with clinical applications. Her group studies phage–antibiotic synergy, which has shown efficacy in urinary tract infection (UTI) models. For example, to optimize therapy for UTIs, phage infection of a biofilm was tested in a laboratory bladder setup complete with flowing artificial urine. These types of environmental studies, coupled with a high-throughput phage discovery and genomics platform, have enabled the lab to design and implement successful phage therapy interventions for patients with UTIs. She noted that successful clinical translation will require parallel progress in regulation, manufacturing, and ecological understanding.

### Oral Abstracts

### A Synthetic Cell Phage Cycle

Paul Soudier, French National Institute for Agriculture, Food, and Environment (INRAE), described reconstituting the T7 phage life cycle within synthetic cells—lipid vesicles containing transcription machinery, translation machinery, and engineered *E. coli* receptors. His team demonstrated DNA injection, replication, and partial lysis in these artificial systems, offering a controllable model for dissecting viral replication and designing synthetic phage platforms.

### Cell-Free Production of Infectious Phages from Isolated DNA

Hayley Nordstrom, PhD, J. Craig Venter Institute, demonstrated that cell-free transcription–translation systems can generate infectious phages directly from DNA templates. Using *Klebsiella* phages, she showed how simple, one-pot reactions can efficiently produce viable phage particles within hours. Optimization of the quality and amount of template DNA, ensuring good oxygenation, minimizing evaporation and condensation, and tuning the incubation time are critical factors for success. The approach offers a fast, automatable alternative for phage boot-up and screening during development.

### *De Novo* Synthesis, Golden Gate Assembly, and Rebooting of Mycobacteriophage

Ching-Chung Ko, PhD, University of Pittsburgh, presented a modular Golden Gate Assembly strategy for building synthetic mycobacteriophages that facilitates the reconstruction of phage genomes from individual synthetic fragments. Only a small subset of the large number of sequenced mycobacteriophages has so far proven clinically useful. To lay the foundation for creating a broader phage library, Dr. Ko designed, assembled, and rebooted 2 synthetic mycobacteriophages and several derivatives thereof. He used Golden Gate Assembly to create genomes from around a dozen fragments, then electroporated them into *Mycobacterium smegmatis* to reboot the phages. This was particularly noteworthy because of the high G+C% content of the fragments, which were synthesized using TdT chemistry, and the large genome sizes (~41 kb and ~50 kb). In addition, future desired changes to make the genomes more useful, such as gene deletions, insertions, and modifications, can be made at the fragment/plasmid level, and novel phages then rebooted and potentially used clinically. This method enables rapid, precise engineering of therapeutic phages and customized host-range mutants.

### Engineering Phage Quasispecies Using Machine Learning to Break Through Evolutionary Barriers

Phil T. Huss, PhD, University of Wisconsin–Madison, noted that naturally occurring host-range mutants can be powerful tools to broaden a phage’s therapeutic potential. But phage quasispecies are dominated by wild-type phages, and the likelihood of finding a host-range mutant that requires more than 2 genomic changes is remote. To create a more diverse and useful phage quasispecies, Dr. Huss first trained a machine-learning model on data related to host range, then used it to design an extensive library of mutations in T7 that were likely to drive host-range changes. After using ORACLE to produce this library, it was tested on clinically relevant pathogens and valuable host-range mutants were identified. This approach to engineering targeted variation into a quasispecies may accelerate the ability to identify useful mutants and apply them clinically.

### Engineering Phages for Serotype-Specific Targeting of *E. coli*

Pallavi Raj Sharma, PhD, Gladstone Institutes, outlined that many pathogenic *E. coli* strains have O-antigen layers that present a challenge to targeted phage therapy. In order to design phages that specifically target pathogenic *E. coli* strains, Dr. Sharma mined gut-associated *E. coli* genomes, creating a database matching the tail spikes of prophages with the serotype of the strain in which they were found. Leveraging this information, she demonstrated that phages engineered with chimeric tail spikes could target a desired *E. coli* serotype, opening the door to using the targeting database for specific and precise clinical applications.

Valery Ortiz, Scripps Research Institute, presented cryo-electron tomography analyses of the interactions of mycobacteriophage Fionnbharth with *M. smegmatis*. Using cryo-electron microscopy and Focused Ion Beam milling (cryo-FIB) to create ultra-thin electron-transparent slices (lamellae), phage-host complexes could be visualized in their native environment. A pre-binding state shows an extended tail tip complex searching the cell surface with the central tail fiber. Upon binding, the tail tip complex flattens tightly against the cell surface, and the tail tube aligns perpendicular to the cell.

### Symposium: Bacteriophage Engineering, Synthetic Biology, and Advancing Therapeutic Utility

### Synthetic Phage Genomes: Assembly and Rebooting

Erica Hartmann, PhD, Northwestern University, shared her recent work on projects related to studying hidden phage diversity in the environment. Metagenomic sequencing has provided a wealth of novel phage genomic information, but without access to the correlated phage particles. She discussed her efforts using genetic sequencing to identify phages in locations such as toothbrushes and built environments. While traditional plaque assays require culturable hosts and have major limitations, her work demonstrates that sequencing-based approaches can now reveal vast, previously hidden phage diversity in many environments, though genome annotation and functional validation remain challenging. She presented work seeking to bridge this gap by taking steps toward the goal of rebooting phage genomes found in metagenomic samples. To do so, she used yeast to assemble a synthetic genome of the clinically useful *P. aeruginosa* phage JG024 (~66 kb), then rebooted it in multiple *P. aeruginosa* strains. She noted that successful and efficient rebooting requires knocking out host defense systems, an important finding for potentially higher-throughput future applications. She described work producing functional *P. aeruginosa* phages in non-native yeast hosts, “phage rebooting,” to study and engineer phages for new applications. Her talk explored how sequencing and synthetic biology can expand understanding of phage biology and open avenues for innovative biotechnology approaches.

### Systematic High-Throughput Characterization of Phage-Host Interactions to Build Predictive Models of Phage Susceptibility

Vivek Mutalik, PhD, Lawrence Berkeley National Laboratory, presented high-throughput work by the Phage Foundry team aimed at elucidating the mechanisms driving phage–host interactions. In addition to a repository of genetically diverse and clinically relevant antimicrobial-resistant bacterial pathogen strains, Phage Foundry has also built an interaction matrix of phage-*Pseudomonas* containing 9,500 interactions mapped in triplicate, which can be interrogated to predict phage susceptibility. This dataset can also be used to identify the genetic basis of host sensitivity and has already been used to identify receptors for hundreds of phages. These large data sets on essential genes, phage/host interactions, phage receptors, and host factors can be used to train machine-learning artificial intelligence (AI) models toward eventually being able to predict a phage cocktail for effective control, given a novel pathogen’s genome sequence.

David Gally, PhD, University of Edinburgh, presented work on using machine learning to identify or design effective phages using genomic data and large datasets of known phage–bacteria interactions. He described studies using 2 approaches: comparing bacterial genomes to known phage-sensitive strains and matching phages based on biological features such as receptors or defense systems. In a proof-of-concept study, he reported that 314 uropathogenic *E. coli* isolates were tested against 30 phages to train Random Forest models that predict phage efficacy from bacterial gene content. This approach was found to inform bespoke phage selection and identification of key genetic determinants for therapeutic success. The models can then be interrogated to yield insights into what phage and host factors are most critical in assessing susceptibility or resistance, and that information can be used to design customized and effective phage cocktails for treatment.

### Poster Presentations

### M1: Preclinical purification, stabilization, and formulation

This poster session included abstracts that described advances in phage production, purification, and formulation.

Arik Makovitzki, Hadassah Medical Center, shared a practical solution to one of phage therapy’s biggest challenges—purification. His team replaced the cumbersome, low-yield traditional methods with a 2-step monolithic chromatography process that quickly and cleanly removes bacterial contaminants while preserving phage activity.

Jessica Sacher, PhD, Stanford University, shared a simple but powerful way to clean up phage preparations using a single-step OH-1 monolithic chromatography process. Her method worked across different phage types, stripping away nearly all endotoxins and proteins while keeping most of the phages intact. Impressively, even large and complex phages purified well, and the cleaned samples met standards for clinical use. The approach is fast, reliable, and easy to repeat.

Marie Komárková, PhD, Masaryk University, presented a study on how different antimicrobial preservatives affect the stability of therapeutic phages. Since preservatives are needed for multidose formulations, her team tested 12 common preservatives to see which could safely coexist with *Staphylococcus Kayvirus* and *Pseudomonas Pbunavirus*. The 2 phages were stable in most preservatives, but several, including benzalkonium chloride, chlorhexidine, and diazolidinyl urea, completely destroyed phage activity. Others, including m-cresol, sodium propionate, sodium benzoate, and phenylethyl alcohol, caused only mild titer drops, while parabens, thimerosal, and monopropylene glycol kept the phages stable for weeks. Her results offer practical guidance for designing phage formulations.

Amelia Schmidt, PhD, Stanford University, introduced an inventive approach to fighting dental plaque by combining bacteriophages with nanoparticles. Her team attached *Streptococcus mutans*-targeting phages to gold and chitosan nanoparticles, aiming to help them penetrate tough biofilms that normally block treatments. The hybrid “phage-nanoparticle” design is expected to boost killing power, extend retention on tooth surfaces, and allow future delivery of imaging or therapeutic agents.

Shruti Sawant, Purdue University, presented an innovative approach to making inhaled phage therapy more effective for recalcitrant lung infections. Her team used liposomes to reduce loss of activity in the nebulization and delivery processes and to reduce pulmonary cellular uptake to enhance airway retention of phages. The liposome-encapsulated phages stayed active longer, reached deeper into the lungs, and were released slowly over time.

Tony Zhou, PhD, Purdue University, presented a new dry powder aerosol bacteriophage formulation designed for lung delivery. The dry powder formulation produced fine, easily inhaled particles and retained potency for >3 months at 4 degrees Centigrade, suggesting a practical, shelf-stable way to deliver phage therapy.

Qingquan Chen, PhD, Stanford University, presented HydroPhage, a soft hydrogel that slowly releases bacteriophages over the course of a week. This practical, sustained-release system is designed to make topical phage therapy more consistent and potent, offering a promising new approach for treating drug-resistant wound infections.

Austen Terwillinger, PhD, Baylor College of Medicine, described the launch of TAILΦR, the first academically funded Good Medical Practice facility in North America dedicated to personalized phage manufacturing. The center can rapidly produce customized phage cocktails, enabling clinicians to respond more quickly and effectively to evolving drug-resistant infections.

### M2: Preclinical Bacteriophage Cocktails and Bacteriophage-Antibiotic Synergy

This session featured a dozen poster presentations that outlined novel approaches to the rational construction of phage cocktails and to the exploitation of phage–antibiotic synergy.

Madison Stellfox, MD, PhD, University of Pittsburgh, described a broad phage cocktail for the treatment of Enterococcal infections. Her team created a 6-phage combination with strong activity across diverse clinical isolates. The cocktail was active against 11/11 *E. faecalis* but only 14/31 *E. faecium* clinical isolates referred for evaluation. This approach could significantly shorten the time from request to treatment for compassionate-use phage therapy.

Matthew Imanaka, National Institute of Infectious Diseases, Tokyo, presented the development of a 5-phage cocktail designed to target carbapenem-resistant *Enterobacter cloacae* complex strains circulating in Japanese hospitals. Starting with 3 phages that covered most isolates, his team added 2 more to overcome resistant types, achieving 94% coverage across 133 clinical strains.

Luke Sobolewski, Locus Biosciences, presented an automated platform that uses robotics and computer vision to rapidly screen and optimize phage cocktails. The system can test hundreds of phages against many bacterial strains at once, producing consistent, scalable data.

Shiman Guo, University of Hong Kong, developed a phage cocktail targeting carbapenem-resistant *Klebsiella Pneumoniae* (CRKP). The team isolated 500 CRKP strains and matching phages from wastewater across Hong Kong, analyzed their genomes, and selected 4-6 potent lytic phages with broad and complementary anti-CRPK activity. The cocktail successfully reduced CRKP infection in mice.

Conner Kovacs, Locus Biosciences, described how his team collected samples from across the US to build a library of over 8,000 phages. Using robotics with advanced data analysis to assess antibacterial activity, they measured over 41 million phage-bacterial interactions. These data were provided to a “cocktail calculator” to predict optimal phage combinations, thereby providing a short turnaround platform to create targeted treatments for drug-resistant infections.

Lika Leshkasheli, PhD, Eliava Institute, shared how her team has been successfully using bacteriophages to treat antibiotic-resistant *Klebsiella* infections. The institute has built one of the world’s largest collections of *Klebsiella* phages, which they use to develop both ready-made cocktails and personalized treatments for individual patients. She described clinical cases that demonstrated the Eliava Institute’s integrated phage therapeutic approach for the treatment of difficult bacterial infections.

Alexander Bobrov, MD, PhD, Walter Reed Army Institute of Research, described efforts at the Institute to develop phage cocktails that are effective against *P. aeruginosa* or *S. aureus*. Phages were isolated from environmental sources and combined into cocktails designed for broad anti-microbial activity. Five broadly active phages were combined into a bivalent cocktail that covered 83% of a *P. aeruginosa* diversity panel and 89% of a *S. aureus* diversity panel. The bivalent cocktail (designated PS1) demonstrated substantial clinical efficacy against bimicrobial staph/pseudomonas infections in murine infection models.

Emma Kane, Yale University, showed that pairing the phage OMKO1 with ceftazidime, aztreonam, colistin, or tobramycin greatly enhanced the killing of drug-resistant *P. aeruginosa*, even at low antibiotic doses, highlighting the potential of phage–antibiotic combinations to strengthen treatment against resistant infections.

Ran Nir-Paz, MD, Hadassah Medical Center, showed that *in vitro* phage regrowth patterns can help predict *in vivo* treatment outcomes and guide smarter phage–antibiotic dosing strategies. His team found that phages with slower regrowth *in vitro* achieved stronger and more durable bacterial clearance in mice when combined with ceftazidime, while pairing with amikacin led to antagonism.

Jin Maneekul, PhD, University of Pittsburgh, presented research on phage-encoded lysterase enzymes as potential treatments for difficult *M. abscessus* infections. Her team tested 6 lysterases from different mycobacteriophages and found that all could kill mycobacteria in a dose-dependent way, including many clinical *M. abscessus* isolates. The enzymes also worked synergistically with amikacin, enhancing bacterial killing.

Thomas Smytheman, Seattle Children’s Research Institute, recognizing that persons with HIV infection are at risk for mycobacterial infections, studied whether commonly used antiretroviral and antimycobacterial drugs adversely affected phage activity against *M. abscessus*. Most phage and antituberculous drug combinations showed no interference, though bedaquiline displayed some antagonism with certain phages. Tests with antiretroviral therapy (ART) drugs (dolutegravir, tenofovir, lamivudine) showed only minimal interactions with phage activity. These studies provided reassurance that phages can be used for the treatment of mycobacterial infections in people with HIV infection.

Rajnikant Sharma, PhD, University of Southern California, studied how different nutrient environments and timing of antibiotics to phage–bacterial cultures affect phage activity against *P. aeruginosa in vitro*. His team found that both the composition of nutrients and the relative timing of phages and antibiotics to bacterial cultures affected the extent of phage-antibiotic synergy. The study highlights the complexity of extrapolating *in vitro* results to clinical scenarios in which growth conditions and nutrient milieu may significantly vary from those present in *in vitro* study conditions.

### M3: Other Topics in Preclinical Research

Session M3 consolidated posters focused on miscellaneous preclinical research topics.

Baptiste Bourgine, PhD, Precise Health SA, presented an AI-based “digital phagogram” system (the Digital Phagogram Platform) that can identify effective bacteriophages for multidrug-resistant infections within hours. The platform, called PhageMatch, analyzes bacterial genome data using AI and finds compatible phages from global phage banks. Validating its predictions *in vitro* and in infection models using *Galleria mellonella* larvae and mice, the team demonstrated that the AI-selected phages effectively killed multidrug-resistant *E. coli* strains.

Dallas Mould, PhD, Yale University, examined whether calcium concentration might be a variable that contributes to discordant results when phages with antimicrobial activity *in vitro* fail in the clinic. Since many patients receiving phage therapy are hypocalcemic, phage activity may be compromised compared to that demonstrated *in vitro*. In a large screen of *P. aeruginosa* phages, about 42% required calcium for potent antibacterial activity. Genomic analysis showed that calcium-dependent phages had more calcium-binding domains, offering a possible way to genotypically predict this trait. In one clinically tested phage, TIVP-H6, limited calcium hindered replication after initial infection steps. Supplementing calcium in sputum samples from cystic fibrosis patients restored phage activity, while calcium-binding molecules and other microbes could block it.

Blake Jackson, University of Pittsburgh, reported the successful induction of prophages from clinical isolates of *Burkholderia cepacia* complex. Five of these phages and a lytic phage previously used for the treatment of a patient with a *Burkholderia cepacia* complex infection were tested against 40 clinical strains of *Burkholderia*. Thirty-two of these *Burkholderia* strains were susceptible to at least 1 of the 6 phages. Phage activity was negatively affected by nebulization.

Eric Nelson, MD, PhD, University of Florida, studied how bacteriophages that attack *Vibrio cholerae* can interfere with rapid diagnostic tests (RDTs) used during cholera outbreaks. His team analyzed samples from over 2,000 patients across Bangladesh and found that when these phages were present, RDT sensitivity often dropped—especially in patients with severe dehydration, likely because the phages reduced detectable bacterial levels. Interestingly, when *V. cholerae* carried phage-resistance genes, test accuracy improved significantly.

### M4: Clinical Applications

Session M4 included 7 poster presentations highlighting clinical experiences with phage therapy.

Keiko Salazar, PhD, Baylor College of Medicine, reviewed the TAILOR Laboratory experience delivering personalized phage therapy to patients with severe, drug-resistant infections. Since 2019, the team has received nearly 500 requests for phage treatment and screened over 450 bacterial isolates from more than 200 patients, covering over 20 different bacterial species. From this work, they built a library of more than 360 phages active against major antibiotic-resistant pathogens. Among 30 patients who ultimately received phage therapy under compassionate-use protocols, 21 achieved bacterial eradication, though 6 experienced relapse within 3 months.

Mariam Dadiani, MD, of the Eliava Phage Therapy Center, reviewed the Center’s clinical experience with personalized bacteriophage therapy. Between 2018 and 2025, Eliava treated patients with customized phage cocktail designed by phage susceptibility testing. Treatments were delivered orally, topically, by inhalation, or via localized application, depending on the infection site. Most patients showed marked improvement or complete recovery, particularly those with urinary and respiratory infections.

Hamza Hajama, San Diego State University, investigated how *Achromobacter xylosoxidans* in cystic fibrosis evolves during phage therapy in patients treated with phage cocktails alongside antibiotics and closely monitored how both *Achromobacter xylosoxidans* and patient microbiomes responded over time. The team found an 80% to 90% drop in *Achromobacter* DNA in sputum and blood after treatment, with these reductions lasting for months. Interestingly, some bacterial isolates that developed phage resistance also became more sensitive to antibiotics, suggesting an evolutionary tradeoff.

Gail Stanley, MD, MPH, Yale University, studied personalized inhaled phage therapy in people with cystic fibrosis who are chronically infected with multidrug-resistant *P. aeruginosa*. Nine patients with cystic fibrosis received nebulized phages targeting *Pseudomonas* surface structures lipopolysaccharide and type IV pilus. Patients showed marked clinical improvement, and treated bacterial isolates showed less attachment and biofilm formation. Overall, the study shows that phage therapy not only kills multidrug-resistant bacteria but also weakens their virulence and reduces lung inflammation. These findings support the concept of a “trade-off ” strategy.

Ritwik Kumar, UC Irvine, explored an intranasal phage therapy approach to treat MRSA sinusitis by intranasal irrigation to deliver a combination of a custom phage (ΦLudmila), isolated from Southern California wastewater, and IV vancomycin. The patient reported improved sinus symptoms and less congestion with no side effects. Cultures from sinus rinse samples showed no detectable methicillin-resistant *S. aureus* by week 2 of therapy. ΦLudmila phage was shown to infect two-thirds of tested *S. aureus* strains, including both MRSA and methicillin-susceptible *S. aureus* types.

Marisa Azad, MD, PhD, Ottawa Hospital, reported the use of phage therapy to successfully treat a periprosthetic joint infection caused by multidrug-resistant *S. epidermidis*. The patient had experienced 10 previous hip surgeries but had persistent infection despite multiple rounds of antibiotics, including a prolonged course of daptomycin. Phages were delivered both intra-articularly and intravenously, twice daily for 14 days, which resulted in a full healing of the surgical wound and significant improvement in the patient’s pain and mobility. At 10 months post-treatment, she remained infection-free with no need for further surgery.

Tobi Nagel, PhD, Phages for Global Health, is working with colleagues to develop a National Plan for Phage Therapy in Malaysia in response to the rising crisis of antimicrobial resistance in the region. Malaysia faces alarming rates of drug resistance, with 65% of *Acinetobacter baumannii* bloodstream infections resistant to carbapenems and hospital mortality exceeding 50%. Recognizing the urgent need for alternatives, the Malaysian Ministry of Health launched the initiative in 2025. The program could serve as a blueprint for other countries, demonstrating how countries with high antimicrobial resistance burdens can adopt phage therapy as a sustainable, locally driven solution to antibiotic resistance.

### M5: Other applications

This session included posters that illustrated the use of phages to deliver tailored antigens as an innovative vaccination strategy, as well as ways in which phages could be used to shape the gut microbiome and used for diagnostic purposes.

Chelsea Stamm, PhD, Catholic University of America, described the use of engineered T4 capsids to deliver payloads to hematopoietic stem cells. The Hoc and Soc capsid decoration proteins were used to display stem cell-specific mAbs and payload proteins, respectively. A cationic lipid coating was added to improve transduction efficiency. By packaging the capsid full of engineered DNA with mammalian expression cassettes, this platform could deliver both protein and genomic payloads to human stem cells.

Ayobami Awe, Catholic University of America, shared work on developing a flu vaccine based on bacteriophage T4. The capsid decoration proteins Hoc and Soc were fused to conserved antigenic regions of the influenza virus: the M2e domain and the HA stalk. The Hoc-3M2e fusion was created with genomic engineering of the T4 genome and displays M2e domains from human, swine, and avian influenza. The Soc-HA stalk fusions used Spycatcher technology to bind capsids to purified HAs expressed in mammalian cells.

Jarin Taslem Mourosi, Catholic University of America, reported on the development of a bacteriophage T4-based vaccine for the dengue virus. This platform displays an antigenic segment from the dengue virus envelope on the T4 capsid via a fusion to the Soc decoration protein. Antigens from the 4 serotypes of dengue can be simultaneously displayed, and expression of the fusions can be increased by tuning promoters.

Kosuke Fujimoto, PhD, Osaka University, reported on an enzyme derived from an *Enterococcus* phage that lyses *E. faecalis* biofilms. A therapeutic application of this enzyme is in the prevention of *E. faecalis* expansion in the intestines, a condition associated with acute graft-versus-host disease in allogenic hematopoietic cell transplant patients. Studies on mice with graft-versus-host disease suggest enzyme treatment to control *E. faecalis* growth can decrease bacterial burden and increase survival time.

Kesia da Silva, PhD, Stanford University, described a colorimetric phage-based assay for the surveillance of the human pathogen *Salmonella typhi*. The approach costs less than the traditional plaque assay and delivers rapid results on the timescale of the far more expensive PCR tests. Additionally, it is stable for months at room temperature and requires little manual labor, making it scalable and optimal for use in the field.

Roshan Nepal, PhD, CSIRO Aquaculture, described the use of phages as tools for pathogen detection in water and their use as antimicrobial agents in the treatment of water. Sampling tiger prawns revealed 13 *Vibrio* species, and bacteriophages that infect these species were isolated. A cocktail of these phages could be used to control *Vibrio* populations in closed prawn hatcheries.

### M6: Bacteriophage Pharmacokinetics and Pharmacodynamics

This session provided further insights into the challenges inherent in phage susceptibility testing and in the rational design of phage–antibiotic combinations.

Gauri Rao, PharmD, University of Southern California, observed that exposure of *P. aeruginosa* to a cell lysate from *P. aeruginosa* but not other bacterial species results in a reduction of susceptibility to phage infection. This process requires the presence of a 2-component regulatory system (Gac/Rsm). The component of *P. aeruginosa* responsible for the downregulation of susceptibility is present in organic extracts of cell lysate but has not yet been identified.

Nicholas Smith, PharmD, PhD, University at Buffalo, SUNY, studied the effect of varying the phage inoculum size in spot test assessments of phage susceptibility of 135 multidrug-resistant and extensively drug resistant clinical isolates of *P. aeruginosa* to 3 clinically relevant phages (E215, PACE, and PY02). The investigators demonstrated that interpretation of phage susceptibility tests in this spot test-based system were significantly affected by phage inoculum size. At high concentrations, phages were judged to be active against 89 of 135 strains. When lower inocula were used, the imputed phage susceptibility declined to 39 of 135 strains. The study demonstrated one of the important variables that will be critical to control as phage susceptibility testing is further standardized.

Gauri Rao, PharmD, University of Southern California, presented a stepwise approach to the design of optimal phage-antibiotic combination regimens for the treatment of *P. aeruginosa* infections. The approach assesses phage efficiency of plating against the target bacterial strain. Phages with efficiency of plating >0.75 are studied for their ability to reduce minimum inhibitory concentration of clinically relevant antibiotics in static concentration time-kill assays. After defining key phage characteristics (burst size, absorption rate, and latent period), the most effective combinations are studied in hollow fiber infection models. This systematic approach to determining optimal combinations of phage and antibiotics warrants further study and clinical correlation.

### M7: Bacteriophage Ecology: Bacteriophage and the Microbiome

Session M7 featured posters that outlined how environmental and host community factors influence phage biology and antimicrobial activity.

Leisl K. Jeffers-Francis, PhD, North Carolina State and Technical University, reported that *E. coli* exposed to phage T4 and T7 and iron stress evolved resistance to both phage and iron toxicity through mutations in lipopolysaccharide biosynthesis genes, redox-detox regulators, and transcriptional regulators.

Kate B. R. Carline, College of William and Mary, demonstrated that engineered *M. smegmatis* and phage Kampy persist in sterilized and non-sterilized soil microcosms but only spread in some conditions. Higher prevalence of Kampy was observed in sterilized soils.

Katherine K. Ennis, PhD, University of California Berkeley, showed that phages can negatively affect the growth of phylogenetically distant non-host bacteria. Phage sensing by non-host bacteria occurs through sensor kinases and response regulators and incurs a fitness cost.

Jyot Antani, PhD, Yale University, exposed *E. coli* to flagellotropic phage Chi in swim plate assays that select for motility and reported the emergence of early non-motile mutants followed by motile mutants with mutations in *fliC*. Some of these motile mutants showed increased motility compared to the ancestor.

### M8: Bacteriophage Screening, Discovery and Optimization

Posters presented in this session described efforts to expand phage libraries through environmental screening and *in vitro* training.

Adeline Supandy, PhD, University of Pittsburgh, reported that 10 *Klebsiella* phages, including jumbo types, revealed 2 resistance mechanisms—capsule loss and lipopolysaccharide modification. Despite large genomes, jumbo phages had narrow host ranges. Combining capsular polysaccharide- and lipopolysaccharide-targeting phages may enhance therapeutic coverage.

Ortal Yerushalmy, PhD, Hebrew University, reported isolation of 76 novel *Klebsiella* phages. These phages covered 71% of *Klebsiella* isolates in an assembled archive of *Klebsiella* strains; 2 were jumbo phages, and several showed synergy with antibiotics, supporting phage–antibiotic combination therapy.

Sarika Bapat, University of Pittsburgh, isolated phages from wastewater that effectively targeted New Delhi metallo-beta-lactamase-producing *Citrobacter, Klebsiella*, and *Enterobacter*. These phages may have clinical use in the treatment of patients with plasmid-mediated carbapenem-resistant bacterial infections.

Camilla Do, Baylor College of Medicine, used geographical phage mapping (geΦmapping) and a portable phage-hunting device (ΦHD) to achieve rapid identification of pathogen–phage reservoirs and created a library of novel phages targeting resistant bacteria.

Justin Massey, MD, University of Pittsburgh, isolated 8 *Achromobacter* phages; 4 showed broad activity against 76% of clinical isolates. Compassionate-use treatments were safe, supporting upcoming Phase I trials for cystic fibrosis-related *Achromobacter* infections.

Colin M. Lewis, University of Pittsburgh, described the outcome of a phage discovery course in South Africa. In this phage workshop, 25 diverse mycobacteriophages from *M. smegmatis* were isolated, including rare subclusters I2 and A20, highlighting vast, underexplored global phage diversity and the educational value of geographically distributed phage educational programs.

Murray E. White, University of Pittsburgh, deployed the Applemans protocol for directed evolution of *Mycobacterium* phages for infection of challenging strains of *M. abscessus*. Using iterative rounds of evolution, he derived phage Muddy_AR85 with an enhanced ability to infect and kill some smooth *M. abscessus* strains, expanding therapeutic potential for difficult Nontuberculous mycobacteria infections.

Satoshi Uematsu, MD, PhD, Osaka Metropolitan University, used of metagenomic analysis of stool in patients with recurrent *Clostridioides difficile* enterocolitis and revealed that phage–bacteria cooperation played a key role in restoring gut balance. Ten novel *C. difficile* endolysins were identified with potent antimicrobial activity, offering new therapeutic potential.

### T1: Bacteriophage Structure

This poster session included 4 posters that described novel insights into phage structure.

Krista Freeman, PhD, Case Western Reserve University, provided details of the tail tip structures of 2 therapeutic mycobacteriophages, Muddy and BPs. The models have conserved elements like the distal tail, baseplate hub, and central tail fiber proteins, plus unique features that would be difficult to bioinformatically predict. Among these is an example of mycobacteriophage overprinting, which is the expression of 2 distinct proteins from different frames of a genomic region.

Meng-Chiao Ho, PhD, University of Kansas, presented a dissection of the tail tip complexes of mycobacteriophages Claus, Corndog, and Mysterious. The tail tube, distal tail, and baseplate hub proteins in these phages interact similarly, but the receptor-binding proteins surrounding these cores are diverse. Interestingly, the different interactions between the baseplate hub and central tail fiber proteins suggest distinct unlocking strategies for genome release during infection.

Jarin Taslem Mourosi, Catholic University of America, reported an investigation of the portal protein of *E. coli* bacteriophage T4. Deleting the first 3 translated residues reduces expression of the portal protein, disrupting capsid maturation and cleavage. Suppressor mutations in the noncoding region upstream of the portal gene restore expression levels and normal capsid maturation. It is hypothesized that a small RNA in this region may interact with the portal.

Donghyun Raphael Park, PhD, Scripps Research Institute, displayed cryo-electron tomograms of *M. smegmatis* cells infected with and producing progeny virions of mycobacteriophage Fionnbharth. Cryo-FIB milling was used to prepare thin lamellae before imaging enabled high-contrast visualization of infected cells in their native state. These infection snapshots captured mycobacteriophage assembly intermediates, like empty and partially packaged capsids.

Jamie Wallen, PhD, Western Carolina University, shared a structure-driven analysis of mycobacteriophage immunity repressors, with a spotlight on DNA-binding specificity. X-ray crystallographic structures of DNA-bound repressors from cluster A1 and A2 mycobacteriophages reveal a conserved protein fold with 2 DNA-binding domains. Amino acids within the stoperator-binding domain determine the specificity of DNA recognition; mutating these changes immunity.

### T2: Bacteriophage Engineering

Session T2 included 4 poster presentations that described novel approaches to phage engineering.

Sarshad Koderi Valappil, PhD, University of California, Berkeley, noted that the coevolutionary arms race between phages and bacteria is a hot topic, but the frequency of individual phage lineages—some present in very low percentages—over time has not been characterized to a high resolution. To address this, he engineered a non-essential locus in phage lambda to carry 100,000 distinct random barcodes. These barcoded phages were introduced to *E. coli* and allowed to coevolve with their host for more than 30 days, while the relative barcode abundances were tracked using a BarSeq workflow. This provided a high-resolution view of the changing frequencies of all lineages over time, yielding insights into how the phage population develops and responds to evolutionary changes in the host.

Sarah Voss, Johns Hopkins University, presented a poster that made the point that although the presence or absence of specific phage receptors is one determinant of host range, many *S. aureus*-directed phages can adsorb to clinical strains but are subsequently neutralized, scuppering their therapeutic potential. She found that in one clinical strain of methicillin-resistant *S. aureus*, defense against multiple phages occurred mostly via 2 restriction-modification systems and 3 DNA-targeting systems. Seeking mutants that could escape these defenses, she found some that could evade a Gabija defense system, but were then susceptible to AbiK, or were less fit overall. With an expanding data set identifying which phage genes were targeted by each host defense system, she sought to engineer a recombinant phage that would evade most or all host defense through directed evolution.

Wakana Yamashita, National Institute of Infectious Diseases, Tokyo, demonstrated that engineering phages to evade specific bacterial defense systems will likely prove a fruitful way to broaden the application of phage therapy. In that vein, she focused on the little-studied Tmn defense system in *E. coli*. From a group of 7 closely related phages, only one of which could efficiently infect a Tmn-expressing strain, she identified a gene responsible for evading Tmn defense. Engineering this gene into a Tmn-defended strain allowed it to efficiently infect *E. coli*. Tmn escape mutants were also isolated, their mutations mapped to Nmad5, and then those same mutations were introduced into Tmn-defended strains, again allowing infection of the Tmn strain. This work provides 2 potential routes to engineer phages to evade Tmn defense systems.

Adolfo Cuesta, MD, PhD, University of California, San Francisco, made the point that phage therapy for *P. aeruginosa* infections has shown promise, although it has been somewhat less effective with device-associated infections, possibly because of biofilm formation on device surfaces. To enhance efficacy in these cases, Dr. Cuesta engineered a phiKMV mutant with a ~6 kb deletion, allowing ample space in the capsid to include new and useful payloads. He demonstrated that 2 loci in the mutant could accommodate and express transgenes without significant cost to phage fitness or host range. He engineered 2 antimicrobial-peptide-expressing versions of phiKMV and showed they could inhibit the growth of a phiKMV-resistant strain in a mixed culture via orthogonal killing. Next, Dr. Cuesta hopes to add exopolysaccharide hydrolase to the phiKMV payload with the goal of degrading biofilm structural components.

### T3: Bacteriophage Genomics

This session included 9 poster presentations that touched on various aspects of genetic organization, characterization, and classification.

Elizabeth D. Amarh, PhD, University of Pittsburgh, described the genomic and morphological characteristics of 46 curtobacteriophages. She reported that all were dsDNA-tailed viruses, with genomes ranging from 15,138 to 68,074 base pairs and podoviral or siphoviral morphologies. Their genomes group into 5 clusters, some of which include phages that infect other *Actinobacteria*.

Jessie Holsombeck, Lee University, described the cytotoxicity of TChen genes expressed in *M. smegmatis*. They find that 2 genes from the bacteriophage Tchen inhibit the growth of *M. smegmatis* when expressed on a plasmid. These 2 genes are predicted to encode a capsid protein and a minor tail protein.

Sophio Rigvava, PhD, Eliava Institute, reported on the genomic and functional characterization of 2 lytic bacteriophages (LilEfef_55 and EfeBG_143) of *E. faecalis*. They do not encode for virulence or antibiotic resistance genes and have broad lytic activity.

William Myers, Lee University, compared bacteriophages Annapurna and ELee24 from the EF and EE Cluster. He reported on the isolation and sequencing of phages Annapurna and ELee24 on the same plate from the host *Microbacterium foliorum*. Sequencing revealed that they belong to different bacteriophage clusters.

Rebekah M. B. Silva, PhD, New England Biolabs, demonstrated that hypermodification of adenosine in the DNA of the bacteriophage Mu, a process that protects against endonuclease-based phage defenses, is dependent on tRNAGly for the transfer of a glycyl group.

Joseph Stukey, PhD, Hope College, demonstrated that phages had higher fitness if genes linked to the phage reproductive success were downstream of an artificial gene rather than downstream of a non-coding intergenic sequence, which could help drive *de novo* gene creation. These data support the hypothesis that a gene-packed genome enhances phage fitness.

Madison G. Bendele, James Madison University, analyzed a network of gene clusters for phages of *Actinobacteria* to increase our understanding of synteny and mosaicism in phage genomes, which are subject to frequent horizontal gene exchange.

Jemma Fendley, University of California, Santa Barbara, analyzed thousands of actinobacteriophage genomes and found accessory genes grouped in a few locations in the genome, while core gene order is conserved. The study shows a variable linkage disequilibrium between different groups of phages.

Natalie R. Hagen, University of Pennsylvania, explored the “Induce-ome” of *C. difficile*. They characterized phages that could be induced from clinical isolates of *C. difficile*. Many isolates contained more than one prophage, and it was noted that prophages in the same isolate often showed different levels of induction.

### T4: Bacteriophage Host Range

Posters in this session featured work on host range determinants and on approaches to expand host range.

Matthew G. Delach, University of Pittsburgh, isolated 2 host range mutants of phage FionnbharthΔ45Δ47 that gained the ability to kill *M. xenopi*. Both mutants acquired non-synonymous point mutations in a gene encoding for a putative tail spike protein.

Carlos A. Guerrero, PhD, University of Pittsburgh, demonstrated that overexpression of the transcriptional regulator Lsr2 in smooth morphotypes of *M. abscessus* results in a morphotype and phage sensitivity profiles similar to those exhibited by rough strains.

Shengwei Yu, PhD, University of Pittsburgh, reported on the isolation of host–range mutants of *M. chimaera* by screening with high efficiency of plating that show mutations in putative tail spike proteins, as well as other changes in DNA primase, WhiB, Cro-like protein, and helix-turn-helix DNA-binding protein. These host–range mutants infected one or more *M. xenopi* strains that could potentially be used in the treatment of infections with this organism.

Nathan R. Wallace, University of Pittsburgh, demonstrated that *P. aeruginosa* isolates that developed treatment-associated resistance to ceftolozane-tazobactam also showed increased resistance to other antibiotics, while changes in phage susceptibility were variable.

Mariam Elawady, Princeton University, showed that the amplification host can alter phage efficacy. In this system, PA01-amplified phage outperformed PA14-amplified PhiPA3 due to faster adsorption. Amplification hosts can change evolutionary trajectories in long-term experiments.

Wearn-Xin Yee, PhD, University of California, San Francisco, reported on an integrated workflow that facilitates the identification of determinants of *Pbunaviruses* host range. The study showed that the O-antigen is often a physical barrier to adsorption and that its removal restored phage susceptibility. Dr. Yee also identified a new nuclease-based phage defense system.

### T5: Host Defense and Resistance

This poster session focused on topics related to host defense mechanisms and their organization.

Alejandro Mart**í**nez-Calvo, Princeton University, used a 3D imaging platform to study how phages spread through 3D *E. coli* colonies. He revealed that spatially structured *E. coli* colonies that resist or permit phage spread differently from liquid cultures, exposing trade-offs between defense expression and autoimmunity.

Blake Wiedenheft, PhD, Montana State University, studied the structural basis of antiphage defense by an ATPase-associated reverse transcriptase. Structural analysis of a Type I-A retron showed that reverse transcriptases function in phage defense by activating an HNH nuclease that halts protein synthesis and viral replication.

Sara A. Aden, Lehigh University, reported that the cluster N mycobacteriophage Butters encodes gp57r, a prophage defense protein that blocks late-stage infection of other phages, revealing a novel abortive infection-like antiviral mechanism.

Nicole Marino, PhD, University of Pennsylvania, reported that anti-CRISPR AcrVA2 inhibits Cas12a by degrading its mRNA during translation, uncovering a new bacterial gene-regulatory mechanism and tool for programmable CRISPR repression.

Kirsten M. Evans, University of Pittsburgh, reported that phage ATCC 19948-B1, a Kochikohadaviridae member, infected 33% of 12 *E. faecalis* isolates. Resistance correlated with a tagF mutation affecting teichoic-acid synthesis, implicating cell-wall receptors in phage susceptibility.

Andrey A. Filippov, MD, PhD, Walter Reed Army Institute of Research, presented work in which phage-resistant *P. aeruginosa* that were recovered from wounds from phage-treated mouse wound infections were used to isolate phage that were active against these variants from sewage. These were characterized and combined into cocktail PAM5 that was effective against 87% of multidrug-resistant isolates and therapeutic in a mouse lung model. The use of phage-resistant pathogens for the isolation and enrichment of novel phages is a viable approach for broadening the activity of therapeutic phage cocktails.

Albert C. Vill, PhD, Yale University, presented on the evolution pathways of jumbo phages grown in *P. aeruginosa* in cystic fibrosis-like environments. Cultivation in cystic fibrosis-like environments limited evolutionary pathways and selected for distinct resistance mutations. Such evolution experiments inform phage selection for clinical applications.

Eric J. Evans, University of Pittsburgh, performed a genomic analysis of phage defense mechanisms in 112 *E. faecium* isolates. This work revealed diverse antiphage defense systems. Forty-five percent (81) of the 179 putative defense systems were located on the main chromosome, and 98 (55%) were located on mobile genetic elements. Twenty-two of the 98 defense systems located on mobile genetic elements were found on plasmids encoding vancomycin resistance. Analysis of individual defense systems and phage susceptibility showed no apparent correlation. This work highlights the complexity of phage resistance systems among *Enterococci* and raises significant concerns about their horizontal mobility.

Theresa J. Astmann, University of Pennsylvania, reported that the *K. pneumoniae* defense system GAPS1 protects against many phages. Structural modeling will be applied to identify phage anti-defense proteins, revealing co-evolutionary dynamics in antibiotic-resistant strains.

Michael J. Lauer, University of Pittsburgh, studied phage resistance mechanisms of *M. abscessus* in a patient undergoing therapy for *M. abscessus* infection. Mutations in fadD23 and greA genes confer differential resistance to mycobacteriophage Muddy variants, revealing distinct adsorption and cell wall-related mechanisms in *M. abscessus* in phage susceptibility and resistance.

Katherine S. Wetzel, PhD, Georgia State University, reported that *M. abscessus* and *M. smegmatis* require MSMEG_5081/MAB_1350 for infection by several phages. Phage mutants that bypass this block have a single amino acid substitution in their predicted tail spike proteins. Phage-resistant mycobacteria with these resistance pathways have not been observed to emerge during therapy, suggesting that reduced fitness may compromise the ability of *M. abscessus* and *M. smegmatis* to readily make use of these mutations *in vivo*.

### Poster Session T6: Bacteriophage Biology

The final poster session covered diverse aspects of phage biology and provided additional insights into mechanisms of phage resistance and phage infectivity.

Lawrence Abad, Princeton University, demonstrated that *P. aeruginosa* gains phenotypic resistance to phages when exposed to *Pseudomonas* cell lysates and showed that this response is dependent on the 2-component regulatory system Gac/Rsm.

Sriharshita Musunuri, University of California, San Francisco, demonstrated that the therapeutic jumbo phage OMKO1 infection of *P. aeruginosa* does not require the OprM antibiotic efflux pump. She reported that OMKO1 relies on the type IV pilus and flagellum to infect P. aeruginosa and is not dependent on the antibiotic efflux pump OprM, which was previously identified as a receptor for OMKO1.

Renata DiDonato, University of Pittsburgh, studied phenotypic diversity and phage susceptibility of *P. aeruginosa* isolated from patients with otitis. She demonstrated that all but one isolate were susceptible to ciprofloxacin and that 77% of isolates were susceptible to a phage cocktail made of PSA07, PSA16 and PSA39.

Ronen Hazan, PhD, Hebrew University, reported on modified protocols using stainless steel washers, crystal violet staining, and extracellular DNA and ATP quantification that allow for better assessment of phage activity in biofilms.

Dominick Faith, Montana State University, demonstrated that resistant isolates *of P. aeruginosa* isolated after exposure to the lytic phage CMS1 displayed a mucoid phenotype and showed mutations in mucA, a regulator of AlgU.

Arya Khosravi, MD, PhD, Stanford University, reported that *P. aeruginosa* exploits its Pf bacteriophage to acquire iron during chronic infection, revealing a novel phage–bacterium partnership and potential therapeutic target in persistent *P. aeruginosa* disease.

Andrew D. Schmidt, University of Pennsylvania, reported that AcrVA2, an anti-CRISPR protein, inhibits CRISPR-Cas nucleases by degrading mRNA of tagged enzymes, enabling programmable downregulation of Cas9 and Cas12f—marking this the first known Cas12f inhibitor.

Anushka B. Tennakoon, University of Southern Mississippi, screened 71 mycobacteriophage Xavia in *M. smegmatis* and identified 20 cytotoxic genes. These genes included phage structural components, Rha-like transcriptional regulators, DNA modification enzymes, immunity repressors, gene regulatory factors, RusA-like resolvases, and HNH endonucleases. This approach revealed potential new targets for phage-based antibacterial strategies.

Margaret S. Saha, PhD, College of William and Mary, identified diverse, conserved antirepressor proteins in novel mycobacteria satellite phages. She showed that they can modulate prophage induction and retention and revealed unexpected regulatory roles and potential tools for phage bioengineering.

Bavesh D. Kana, PhD, University of the Witwatersrand, reported that mycobacterial resuscitation-promoting factors (Rpfs) modulate phage infectivity. Phages MontyDev and Bora showed reduced infection in Rpf-deficient strains, revealing distinct, cluster-specific dependencies on RpfA and RpfB for optimal lysis and replication.

## CONFERENCE CONCLUSION

The 2025 Conference on Bacteriophages showcased how bacteriophage research has matured into an interdisciplinary science bridging genomics, synthetic biology, regulatory science, and patient care. Across talks, recurring themes emerged: collaboration, standardization, and the convergence of engineering with biology. As one participant summarized, phage therapy now sits “where virology meets medicine,” and the momentum toward clinical translation has never been stronger.

## ADDITIONAL CONFERENCE RESOURCES

Video streamed presentations of all oral content are available at https://www.iasusa.org/events/on-demand-2025-bacteriophage-conference/. Abstracts are published in Topics in Antiviral Medicine (https://www.iasusa.org/tam/october-2025/).

